# Multi-locus variable number tandem repeat analysis of 7th pandemic *Vibrio cholerae*

**DOI:** 10.1186/1471-2180-12-82

**Published:** 2012-05-24

**Authors:** Connie Lam, Sophie Octavia, Peter R Reeves, Ruiting Lan

**Affiliations:** 1 School of Biotechnology and Biomolecular Sciences, University of New South Wales, Gate 9 High St, Sydney, New South Wales, 2052, Australia; 2 School of Molecular Biosciences, University of Sydney, Sydney, New South Wales, 2006, Australia

## Abstract

**Background:**

Seven pandemics of cholera have been recorded since 1817, with the current and ongoing pandemic affecting almost every continent. Cholera remains endemic in developing countries and is still a significant public health issue. In this study we use multilocus variable number of tandem repeats (VNTRs) analysis (MLVA) to discriminate between isolates of the 7th pandemic clone of *Vibrio cholerae.*

**Results:**

MLVA of six VNTRs selected from previously published data distinguished 66 *V. cholerae* isolates collected between 1961–1999 into 60 unique MLVA profiles. Only 4 MLVA profiles consisted of more than 2 isolates. The discriminatory power was 0.995. Phylogenetic analysis showed that, except for the closely related profiles, the relationships derived from MLVA profiles were in conflict with that inferred from Single Nucleotide Polymorphism (SNP) typing. The six SNP groups share consensus VNTR patterns and two SNP groups contained isolates which differed by only one VNTR locus.

**Conclusions:**

MLVA is highly discriminatory in differentiating 7th pandemic *V. cholerae* isolates and MLVA data was most useful in resolving the genetic relationships among isolates within groups previously defined by SNPs. Thus MLVA is best used in conjunction with SNP typing in order to best determine the evolutionary relationships among the 7th pandemic *V. cholerae* isolates and for longer term epidemiological typing.

## Background

Diarrhoeal diseases have been and continue to be a cause of mortality and morbidity, especially in developing countries. Of particular note is the disease cholera, a severe watery diarrhoeal disease caused by *Vibrio cholerae*. *V. cholerae* is a diverse species of Gram negative bacilli. Serological testing has enabled strains of *V. cholerae* to be divided into over 200 serogroups based on the O-antigen present [1]. However, only the O1 and O139 serogroups have been known to cause pandemic and epidemic level disease [2].

Since 1817, seven pandemics of cholera have been recorded [3]. The ongoing epidemic started in 1961 and has affected almost every continent, particularly countries of Southeast Asia, Africa, and South America. Cholera remains endemic in developing countries and outbreaks still pose a significant public health issue [4].

The developments of DNA based typing methods have allowed epidemiological studies of cholera. Methods such as Pulse Field Gel Electrophoresis [5,6], Amplified Fragment Length Polymorphism [7] as well as population structure studies including Multi-Locus Sequence Typing [8-10] have all been applied to *V. cholerae* isolates. Such methods have all been able to distinguish between environmental and clinical strains of *V. cholerae*[6,8,11], but they have had limited success in drawing evolutionary relationships between 7th pandemic strains. Previously, we investigated the evolution of *V. cholerae* using Single Nucleotide Polymorphism (SNP) analysis and found that 7th pandemic *V. cholerae* isolates could be distinguished into groups with a stepwise accumulation of SNPs. The 7th pandemic SNP relationships were confirmed by a large genome sequencing based study by Mutreja *et al*. [12]. SNP Groups were correlated with the spread of pandemic cholera into Africa and were also able to separate the O139 isolates into a distinct SNP profile [13]. However, further resolution of isolates within each group is required.

Multilocus variable number tandem repeat analysis (MLVA) is a PCR based typing method based on regions of tandemly repeated short DNA sequence elements. Variations in the number of copies of repeat DNA sequences form the basis of differentiation [14]. Recent studies have shown that MLVA is a highly discriminating method for the typing of environmental and clinical isolates of *V. cholerae* and is able to differentiate closely related isolates from outbreak situations [15,16]. In this report, we applied MLVA to isolates spanning the 7th pandemic to further determine the genetic and evolutionary relationships within the 7th pandemic clone and to evaluate the potential of MLVA as a long term epidemiological typing tool.

## Results and discussion

### VNTR variation and discriminative power

The MLVA data of 61 7th pandemic isolates including its O139 derivative and 5 genome sequenced strains from Grim *et al.*[17] are presented as repeat numbers for each locus (Table 1). Additionally, 3 pre-7th pandemic isolates were included for comparison but were excluded from the calculation of diversity statistics below. The 66 7th pandemic isolates were distinguished into 60 MLVA profiles. All MLVA profiles were represented by a single isolate except for 4 MLVA profiles that were represented by 4, 2, 2 and 2 isolates respectively. Two of these profiles belonged to SNP group II and had allelic profile of 9-6-4-7-26-14 and 9-6-4-7-25-13. Note that an MLVA profile is made up of the repeat numbers for the following loci (in order): vc0147, vc0437, vc1457, vc1650, vca0171 and vca0283. The remaining profiles were within SNP group VI and differed at vca0171 by only one repeat, with the profiles 10-7-3-9-(22/23)-11.

**Table 1:** Details of*Vibrio cholerae*strains used and their MLVA profiles*

	**Strain**	**Year**	**Country of Isolation**	**Source**^**%**^	**Original laboratory identification**	**VNTR allele**					
						**vc0147**	**vc0437**	**vc1457**	**vc1650**	**vca0171**	**vca0283**
Pre-7th^$^	M66-2	1937	Indonesia (Sulawesi)	Institut Pasteur		8	6	4	7	14	28
	M543	1937	Iraq	NCTC	5395	6	17	4	7	17	31
	M640	1954	Egypt	NCTC	9420	8	14	4	5	11	24
I	M686	1968	Thailand	AFRIMS	SP-EV-29-1	8	6	4	7	15	21
	M799	1989	Hong Kong	University of Hong Kong	In21	9	6	3	8	16	15
	M803	1961	Hong Kong	Institut Pasteur	HK1	8	6	4	7	11	28
	M804	1962	India	Institut Pasteur	930030	8	5	4	7	15	35
	M805	1963	Cambodia	Institut Pasteur	930059	8	6	4	8	14	24
	M806	1964	India	Institut Pasteur	CRC1106	8	6	4	8	14	35
	M807	1966	Vietnam	Institut Pasteur	601	8	6	4	7	15	19
	M808	1969	Vietnam	Institut Pasteur	1536	9	6	4	7	16	17
	M811	1971	Burma	Institut Pasteur	930029	7	6	4	7	7	33
	M815	1973	Philippines	Institut Pasteur	430035	7	6	4	8	16	21
	M820	1978	Malaysia	Institut Pasteur	EB 251/1MR	7	6	4	7	8	19
	M662	1993	Indonesia (Bali)	State Health Laboratory, Perth	7340	8	7	3	8	12	16
	M663	1992	Indonesia (Bali)	IMVS	2100	7	7	3	8	13	27
	M793	1961	Indonesia	University of Maryland	E9120	8	6	4	7	11	32
	Consensus					8	6	4	7	x	x
II	M812, M817	1971/ 1974	Chad	Institut Pasteur	930046/ 99	9	6	4	7	13	25
	M810	1970	Ethiopia	Institut Pasteur	930038	8	6	4	8	19	23
	M814	1972	Morrocco	Institut Pasteur	113	9	6	4	7	14	27
	M816	1974	Senegal	Institut Pasteur	B998C	9	6	4	7	14	24
	M813, M819	1972/ 1975	Senegal/ Germany	Institut Pasteur	9292/ 232	9	6	4	7	14	26
	M809	1970	Sierra Leone	Institut Pasteur	930037	9	6	4	7	14	25
	M818	1975	Comoros Islands	Institut Pasteur	102	9	6	4	7	15	29
	M821	1982	France	Institut Pasteur	Assous M	12	6	4	7	17	10
	M823	1984	Algeria	Institut Pasteur	Marquez	10	6	4	7	16	17
	M826	1990	Malawi	Institut Pasteur	Bakala Malenge	10	6	3	8	21	21
	M2314	1991	Peru	Instituto Oswaldo, Brasil	348	10	6	4	6	15	11
	M2315	1999	Brazil	Instituto Oswaldo, Brasil	590	8	6	4	6	17	19
	M2316	1998	Peru	Instituto Oswaldo, Brasil	609	8	3	4	5	26	12
	M829	1992	Malawi	Institut Pasteur	F. Francisco	7	6	3	8	18	10
	M830	1993	French Guiana	Institut Pasteur	Modesto	10	6	4	6	19	13
	Consensus					9	6	4	7	x	x
III	RC9†	1985	Kenya			9	6	3	7	26	20
	M650	1976	India	National Institute of Cholera	762/76	8	7	4	8	29	28
	M647	1970	Bangladesh	CCUG	13119	9	7	4	7	14	28
	M795	1976	Bangladesh	University of Maryland	30167	9	7	4	7	18	32
	M797	1986	Hong Kong	University of Hong Kong	V31	9	7	4	7	22	36
	N16961†	1971	Bangladesh			9	7	4	7	23	14
	Consensus					9	7	4	7	x	x
IV	M646	1979	Bangladesh	CCUG	9193	9	7	4	7	20	21
	M822	1983	Vietnam	Institut Pasteur	359	10	7	7	8	17	19
	M764	1989	Thailand	AFRIMS	FX-41-3	7	7	4	5	15	24
	M740	1985	Thailand	AFRIMS	D-145	9	7	4	5	15	25
	M723	1982	Thailand	AFRIMS	WR-32	9	7	4	5	20	22
	M714	1979	Thailand	AFRIMS	96A/CO	11	8	4	8	20	19
	M652	1981	India	National Institute of Cholera	1200/81	9	7	4	8	20	13
	Consensus					9	7	4	x	20	x
V	M824	1987	Algeria	Institut Pasteur	Mekki	8	7	4	8	28	14
	M827	1990	Guinea	Institut Pasteur	Guinea1	8	7	4	8	24	16
	M828	1991	Morrocco	Institut Pasteur	Akretche	8	7	4	8	23	17
	M791	1991	Thailand	AFRIMS	CX-043-0	8	7	4	8	12	20
	MJ1236†	1994	Bangladesh			8	7	4	8	12	19
	CIRS-101†	2002	Bangladesh			9	3	3	9	16	11
	B33†	2004	Mozambique			8	7	4	8	11	20
	M654	1991	India	National Institute of Cholera	413/91	7	7	4	8	15	20
	Consensus					8	7	4	8	x	x
VI	M834	1993	Bangladesh	ICDDR	A25365	10	7	3	8	22	11
	M833	1993	Bangladesh	ICDDR	A24698	9	7	3	9	23	11
	M985, M984, M988, M831	1992/ 1993	India/ Bangladesh	ICDDR	F642/F641/ F657/ A26094	10	7	3	9	23	11
	M987	1992	India	ICDDR	F638	10	7	3	9	23	12
	M989	1993	India	ICDDR	2412-93	10	7	3	9	22	13
	M986	1992	India	ICDDR	F643	12	7	3	9	23	11
	M835	1993	Bangladesh	ICDDR	A25080	10	7	3	9	24	12
	M537, M542^#^	1993	India/ Bangladesh	ICDDR	SK556/ F653	10	7	3(4)	9	23	13
	M545	1993	India	ICDDR	MO229	10	7	3	9	21	13
	MO10†	1992	India			10	7	3	9	22	12
	Consensus^#^					10	7	3	9	23	x

^*^MLVA profile is made up of the repeat numbers (also as allele designations) for the following VNTR loci (in order): vc0147, vc0437, vc1457, vc1650, vca0171 and vca0283.

^$^Pre-7th: pre-seventh pandemic isolates. All other isolates are 7th pandemic (I-V) or its derivative O139 (V) isolates. The roman numerals (I-VI) denote SNP groups as described in Lam et al. [12].

^†^Data for these strains were from published genome sequences. Note that no VNTR data for the recently sequenced Haitian isolates and Peru isolate C6706.

The level of variation differed across the six VNTRs analysed. In total, 7, 6, 3, 5, 19 and 24 alleles were observed for vc0147, vc0437, vc1457, vc1650, vca0171 and vca0283 respectively. It is also interesting to note that the 2 most variable VNTRs are located in the small chromosome while the other 4 less variable VNTRs are on the large chromosome. Additionally, one isolate (M542) amplified two products that differed by one repeat for vc1457 which has been observed previously [16]. However, for phylogenetic analysis and scoring of alleles, only the fragment with the strongest signal was recorded. This VNTR is located within the cholera toxin subunit A promoter region which may have contributed to the decreased variation [18].

The discriminatory power of each VNTR and all 6 VNTRs combined was measured by Simpson’s Index of Diversity (D). The highest D value was 0.957 and was recorded for vca0283. Except for vca0283 and vca0171, all D values were lower than previously reported. Our focus on 7th pandemic isolates which have been shown to be highly homogeneous may have contributed to these lower D values. VNTR vc1457 had the lowest D value of 0.437, which was lower than previously reported (D value = 0.58) [16]. The combined D value of 7th pandemic isolates for all 6 VNTRs in this study was 0.995. We also calculated D values from previous studies by excluding MLVA data of environmental and non-7th pandemic isolates [19-22] and found that the D values were similar and ranged from 0.962 to 0.990 [19-22], when only 7th pandemic isolates were analysed. Analysis using the two most variable VNTRs, vca0171 and vca0283, produced comparable D values, which could potentially reduce the need to use the other markers. This would be particularly useful in outbreak situations where there is limited time and resources available to type isolates. However, typing the isolates in this study using only two loci would not reveal any useful relationships.

### Phylogenetic analysis using MLVA

**Figure 1:**
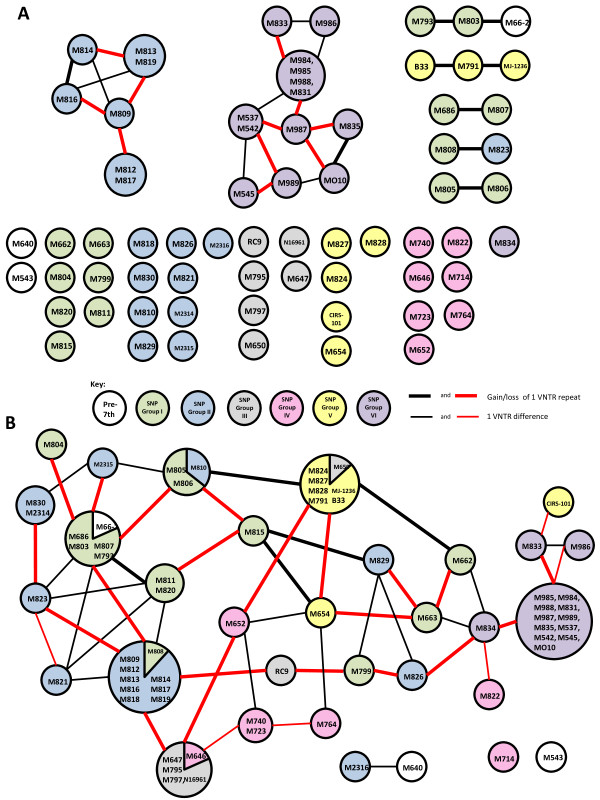
**eBURST analysis and minimum Spanning Networks of 7th pandemic*V. cholerae*isolates based on MLVA.A**) MLVA using 6 VNTR loci and **B**) MLVA using 4 VNTR loci from chromosome I. Each circle represents a unique MLVA profile, with the isolate number/s belonging to the MLVA type within the circles. The colour of each circle denotes the group to which each isolate belongs according to Single Nucleotide Polymorphism (SNP) typing [13] (see Figure 2). Singletons are arranged by SNP groups while members of clonal complexes are connected using minimum spanning network. Thick connecting lines represent differences of one repeat unit with red lines indicating connections chosen in the minimum spanning tree shown in Additional file 1 Figure S 1 based on priority rules described in the text and thin solid lines represent one locus difference with more than one repeat difference. The size of each circle reflects the number of isolates within the circle.

We analysed the MLVA using eBURST [23]. Using the criteria of 5 out of 6 loci identical as definition of a clonal complex, 26 MLVA profiles were grouped into 7 clonal complexes with 37 singletons. For the 7 clonal complexes, a minimal spanning network (MSN) was constructed to show the relationships of the MLVA profiles (Figure 1 A). Many nodes in the 2 largest clonal complexes showed multiple alternative connections. There were 27 possible nodes differing by 1 locus, 4 nodes were due to the difference in vc0147 and 23 others were due to VNTR loci in chromosome II. Out of the 23 single locus difference in the 2 chromosome II VNTRs, the majority (57%) also differed by gain or loss of a single repeat unit. Thus 1 repeat change was the most frequent for the VNTRs on both chromosomes. It has been shown previously that it is more likely for a VNTR locus to differ by the gain or loss of a single repeat unit as seen in *E. coli*[24] and we have also found this was the case in *V. cholerae*. We then used the MLVA data for all 7th pandemic isolates to construct a minimal spanning tree (Additional file 1 Figure S 1A). For nodes where alternative connections of equal minimal distance were present we selected the connection with priority rules in the order of: between nodes within the same SNP group, between nodes differing by 1 repeat difference and between nodes by closest geographical or temporal proximity. The majority of isolates differed by either 1 or 2 loci, which is attributable to vca0171 and vca0283 being the 2 most variable loci. It should be noted that node connections differing by more than one VNTR locus are less reliable as there were more alternatives.

Since the 2 VNTRs on chromosome II were highly variable, exclusion of these 2 VNTRs may increase the reliability of the minimum spanning tree MST (Kendall et al [21]). The number of unique MLVA profiles was reduced from 60 to 32. Nine profiles had multiple isolates, of which 5 contained isolates from 2 different SNP groups. eBURST analysis showed that using only the 4 chromosome I VNTR loci, the majority of the 4-loci MLVA profiles were grouped together as one clonal complex with one locus difference. Two MLVA profiles (represented by M543 and M714) were singletons and another 2 (M640 and M2316) formed a clonal complex by themselves. Out of 37 nodes connected by 1 locus difference, the repeat unit differed by the gain or loss of 1 to 11 repeats. The majority (19 events, 51%) differed by a single repeat unit, followed by 2 and 3 units with 7 and 6 events respectively. Gain or loss of 5 and 11 repeats were only seen in one node each. The MSN for the larger clonal complex showed many alternative connections of the nodes (Figure 1B). Using the same principle as above to resolve alternative nodes with equal minimum distance, an MST was constructed to display the relationships of these MLVA profiles and the 4 more distantly related MLVA profiles as shown in Additional file 1 Figure S1B.

A previous SNP analysis with the same isolates had shown that 7th pandemic cholera had undergone stepwise evolution [13]. None of these groups were clearly distinct from the either the 4 loci or 6 loci MLVA MST aside from SNP group VI which consists of O139 isolates (Figure 1). However, a distinctive pattern can be seen when the consensus alleles within a SNP group are compared as shown in Table 1. We allocated a consensus allele if more than half of the MLVA profiles carried a given allele in the SNP group and if there was no consensus, the consensus allele was represented by an x for discussion below. The 2 most variable VNTRs (vca0171 and vca0283) had no consensus alleles within any of the SNP groups except vca0171 in group VI. The allelic profile that initiated the 7th pandemic was likely to be 8-6-4-7-x-x based on the allelic profiles of the prepandemic stains which is also consistent with the profile of the earliest 7th pandemic isolate M793 from Indonesia. Group I had an 8-6-4-7-x-x allelic profile which evolved into **9**-6-4-7-x-x in group II. By changing the 2^nd^ VNTR allele from 6 to 7, groups III and IV had consensus profiles of 9-**7**-4-7-x-x and 9-**7**-4-x-20-x respectively, with the latter being most likely a 9-7-4-8-20-x profile (see Table 1). Group V had the first VNTR allele reverted back to 8 and had an 8-7-4-8-x-x profile. SNP group VI showed the most allele changes with a 10-7-3-9-23-x profile compared with 8, 7,-, 8, 21/22, 23/16 from Stine *et al.*[15]. Although vca0171 and vca0283 offered no group consensus alleles, it is interesting to note that the trend for vca0171 increased in the number of repeats while vca0283 decreased in the number of repeats over time (Table 1). Each SNP group was most likely to have arisen once with a single MLVA type as the founder, identical VNTR alleles between SNP groups are most likely due to reverse/parallel changes. This has also contributed to the inability of MLVA to resolve relationships. The comparison of the SNP and MLVA data allowed us to see the reverse/parallel changes of VNTR alleles within known genetically related groups. However, the rate of such changes is difficult to quantitate with the current data set.

**Figure 2:**
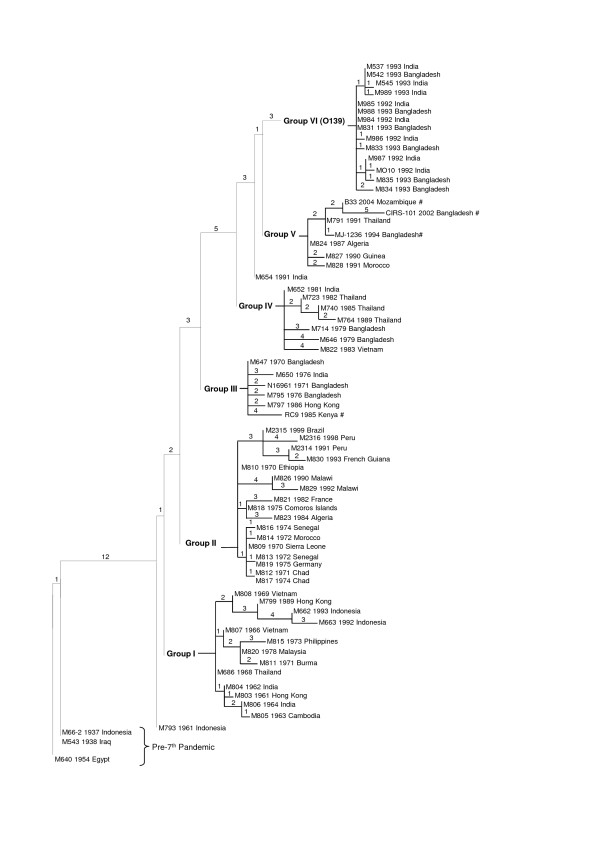
**Composite tree of 7th pandemic*V. cholerae*isolates.** Isolates were separated into six groups according to Single Nucleotide Polymorphism (SNP) typing. Isolates with identical SNP profiles were further separated using Multilocus Variable number tandem repeat Analysis (MLVA). A minimum spanning tree (MST) was constructed for each group and combined with the original parsimony tree. Numbers at the node of each between groups indicate the number of SNP differences, whereas numbers at the node of each branch within a group indicate the number of VNTR differences between isolates.

In order to resolve isolates within the established SNP groups of the 7th pandemic, all 6 VNTR loci were used to construct a MST for each SNP profile containing more than 2 isolates. Six separate MSTs were constructed and assigned to their respective SNP profiles as shown in Figure 2. The largest VNTR difference within a SNP group was 5 loci which was seen between two sequenced strains, CIRS101 and B33. In contrast, there were several sets of MLVA profiles which differed by only one VNTR locus within the MSTs which showed that they were most closely related. The first set consisted of 5 MLVA profiles of six isolates within SNP group II, all of which were the earlier African isolates. The root of group II was M810, an Ethiopian isolate from 1970 which was consistent with previous results using AFLP [7] and SNPs [13]. However, the later African and Latin American isolates were not clearly resolved. We previously proposed that Latin American cholera originated from Africa based on SNP analysis, which was further supported by the clustering of recently sequenced strain C6706 from Peru [25]. Note that C6706 is not on Figure 2 as we cannot extract VNTR data from the incomplete genome sequence. M2314 and M830 from Peru and French Guiana were the most closely related, with 2 VNTR differences, however the remainder of isolates in this subgroup were more diverse than earlier isolates. The second set of MLVA profiles differing by one locus consisted of all O139 isolates in SNP group VI except M834, which was separated by two VNTR loci. This finding is similar to a study by Ghosh *et al*. [26], who found that isolates collected within a year differed at only one locus, while isolates from later years differed at more than one locus. A similar trend was also seen between closely related samples taken from the same household or same individual [21].

Isolates from SNP group V were collected from Thailand and 3 regions of Africa and contained 3 genome sequences, MJ-1236, B33 and CIRS101, from Mozambique and Bangladesh [17]. These isolates were shown to be identical based on 30 SNPs [13]. The genetic relatedness of these isolates was also reflected by their MLVA profiles, which differ by only 2 loci. The consensus alleles for SNP group V was 8, 7, 4, 8, x, x, which was identical to the consensus alleles of MLVA group I (8, 7,-, 8, x, x) according to a 5-loci study by Choi *et al.*[19].

No other consensus alleles of MLVA groups matched the current SNP group consensus alleles. However, there were 2 isolates from Africa (M823 and M826) with the profiles 10, 6, -, 7/8, x, x from this study, which matched 2 MLVA profiles of isolates from MLVA group III Vietnam from Choi *et al.*[19]. These African isolates were collected in 1984 and 1990 while isolates from Choi *et al.*[19] were collected between 2002–2008. It is unlikely that the isolates from these two studies are epidemiologically linked. This further highlights the need for SNP analysis to resolve evolutionary relationships before MLVA can be applied for further differentiation.

Based on a 5-loci MLVA study performed by Ali *et al.*[27] the ancestral profile of the 2010 Haitian outbreak isolates was determined to be 8, 4, -, 6, 13, 36. Nine MLVA profiles differing by 1 locus were found in total and were mapped against our SNP study*.* A previous study showed that 2010 Haitian cholera outbreak strain belong to SNP group V [25]. However, based on the ancestral profile of the Haitian isolates, only the first locus was shared with our group V consensus allele and no other Haitian alleles were found in any of the group V isolates. Thus, no relationships could be made between group V isolates and the Haitian outbreak strains. Similarly, in another 5-loci MLVA study of 7th pandemic isolates sampled from 2002 to 2005 in Bangladesh [21], no MLVA profiles were found to be identical at more than 2 loci to our MLVA profiles. Therefore, while MLVA may be highly discriminatory, it may not be reliable for longer term epidemiology and evolutionary relationships. Our studies of *Salmonella enterica* serovar Typhi also reached a similar conclusion [28]. However, it should be noted that although our isolates are representative of the spread of the 7th cholera pandemic, our sample size is relatively small. A study with a much larger sample may be useful to affirm this conclusion.

## Conclusions

We have shown that MLVA of 6 VNTR loci is highly discriminatory in differentiating closely related 7th pandemic isolates and shown that SNP groups share consensus VNTR patterns. We have also shown that relationships among isolates can only be inferred if they differ by 1 to 2 VNTRs. MLVA is best used for outbreak investigations or tracing the source of outbreaks, such as the recent outbreak in Haiti [27]. The advantage of MLVA is that there is no phylogenetic discovery bias as is the case with SNPs [13]. However, VNTRs alone are too variable to be used for longer term epidemiological studies as they were unable to resolve relationships of the isolates over a 40 year span. MLVA needs to be used in combination with SNPs for evolutionary or longer term epidemiological studies. The SNP and MLVA analyses of the Haitian outbreak and its possible Nepal origin illustrate well the usefulness of this approach [27,29].

## Methods

### Strain selection and DNA extraction

In total, 66 isolates of 7th pandemic *V. cholerae* collected between 1961 and 1999 were used in this study, including 14 isolates of the O139 Bengal biotype (Table 1). Three pre-7th pandemic isolates were also included for comparative purposes. Isolates were grown on TCBS (Oxoid) for 24 hr at 37°C and subcultured for single colonies. Genomic DNA was extracted using the phenol- chloroform method. Where available, VNTR data from sequenced *V. cholerae* genomes was also included in the analysis.

### VNTR selection and MLVA typing

The details of 17 VNTR loci was previously identified and studied by Danin-Poleg *et al.*[16]. Six VNTR loci with D values >0.5 (vc0147, vc0437, vc1457, vc1650, vca0171 and vca0283) were selected and amplified by PCR using published primer sequences which were modified to include a 5’ universal M13 tail as done previously [28]. An additional M13 primer with a fluorescent dye attached was added to the PCR mix to bind to the modified tail. Fluorescent dyes were FAM, VIC, NED and PET for blue, green, black and red fluorescence, respectively.

Each VNTR locus was amplified separately, with each reaction consisting of: ~20 ng DNA template, 2 μM dNTPs, 1 U Taq polymerase (New England Biolabs, Sydney, Australia), 50 μM M13-labelled forward primer, 200 μM M13 primer and 250 μM reverse primer with 2 μl 10 X PCR buffer (50 mM KCl, 10 mM Tris HCl pH 8.8, 1.5 mM MgCl_2_ and 0.1% Triton X-100).

PCR conditions included a touchdown cycling profile as follows: 95°C for 5 min; 96°C for 1 min, 68°C for 5 min (−2°C/cycle, a decrease of 2°C after each cycle) and 72°C for 1 min for 5 cycles; 96°C for 1 min, 58°C for 2 min (−2°C/cycle) and 72°C for 1 min for 5 cycles; 96°C for 1 min, 50°C for 1 min and 72°C for 1 min for 25 cycles; and final extension at 72°C for 5 min.

The fluorescence labelled PCR products of vc0147 (FAM), vc0437 (VIC), vc1457 (PET), vc1650 (NED) in one sample and vca0171 (PET) and vca0283 (NED) in a second sample were pooled for capillary electrophoresis on an Automated GeneScan Analyser ABI3730 (Applied Biosystems) at the sequencing facility of the School of Biotechnology and Biomolecular Sciences, the University of New South Wales. The fragment size was determined using the LIZ600 size standard (Applied Biosystems) and analysed using GeneMapper v 3.7 software (Applied Biosystems). Sequencing was performed to confirm the number of repeats for representative alleles.

### Phylogenetic analysis

A Minimum spanning tree (MST) using pairwise difference was generated using Arlequin v. 3.1, available from http://cmpg.unibe.ch/software/arlequin3, in which if alternative connections of equal distance were present, the connection between isolates with closest geographical or temporal proximity was selected. The Simpson’s Index of Diversity (D value) [30] was calculated using an in-house program, MLEECOMP package [31].

## Authors’ contributions

Experimental work and data collection were carried out by CL. CL, SO and RL contributed to data analysis and interpretation. The study was conceived and designed by RL. The manuscript was drafted by CL and SO, and revised by PR and RL. All authors have read and approved the final manuscript.

## Supplementary Material

Additional file 1 Figure S1.Minimum Spanning trees of 66 *V. cholerae* isolates using MLVA of A) 6 VNTR loci and B) 4 VNTR loci from chromosome I. Each circle represents a MLVA profile, with the isolate number/s belonging to the MLVA type within the circles. The colour of each circle denotes the group to which each isolate belongs according to SNP typing [12] (see Figure 2). If isolates from different SNP groups shared a MLVA profile, the circle was divided to reflect the proportion of isolates in each SNP group. Thick solid connecting lines represent differences of one repeat unit, thin solid lines and dashed lines represent 1 and 2 loci differences respectively, and longer dashed lines represent more than 2 loci differences. The size of each circle reflects the number of isolates within the circle. Click here for file

## References

[B1] Chatterjee SN Chaudhuri K Lipopolysaccharides ofVibrio cholerae. I. Physical and chemical characterization Biochim Biophys Acta 2003 1639 65 79 10.1016/j.bbadis.2003.08.004 14559113

[B2] Reeves PR Lan R Cholera in the 1990s Br Med Bull 1998 54 611 623 10.1093/oxfordjournals.bmb.a011714 10326288

[B3] Barua D Greenough WB Cholera Current Topics In Infectious Disease 1992 Plenum, New York

[B4] WHO Cholera Wkly Epidemiol Rec 2010 85 16

[B5] Dalsgaard A Skov MN Serichantalergs O Echeverria P Meza R Taylor DN Molecular evolution of Vibrio cholerae O1 strains isolated in Lima, Peru, from 1991 to 1995 J Clin Microbiol 1997 35 1151 1156 9114398 10.1128/jcm.35.5.1151-1156.1997 PMC232720

[B6] Cameron DN Khambaty FM Wachsmuth IK Tauxe RV Barrett TJ Molecular characterization of Vibrio cholerae O1 strains by pulsed-field gel electrophoresis J Clin Microbiol 1994 32 1685 1690 7929758 10.1128/jcm.32.7.1685-1690.1994 PMC263762

[B7] Lan R Reeves PR Pandemic spread of cholera: genetic diversity and relationships within the seventh pandemic clone of Vibrio cholerae determined by amplified fragment length polymorphism J Clin Microbiol 2002 40 172 181 10.1128/JCM.40.1.172-181.2002 11773113 PMC120103

[B8] Kotetishvili M Stine OC Chen Y Kreger A Sulakvelidze A Sozhamannan S Morris JG Multilocus sequence typing has better discriminatory ability for typing Vibrio cholerae than does pulsed-field gel electrophoresis and provides a measure of phylogenetic relatedness J Clin Microbiol 2003 41 2191 2196 10.1128/JCM.41.5.2191-2196.2003 12734277 PMC154734

[B9] Salim A Lan R Reeves PR Vibrio cholerae pathogenic clones Emerg Infect Dis 2005 11 1758 1760 10.3201/eid1111.041170 16318732 PMC3367346

[B10] Byun R Elbourne LD Lan R Reeves PR Evolutionary relationships of pathogenic clones of Vibrio cholerae by sequence analysis of four housekeeping genes Infect Immun 1999 67 1116 1124 10024551 10.1128/iai.67.3.1116-1124.1999 PMC96437

[B11] Karaolis DK Lan R Reeves PR Molecular evolution of the seventh-pandemic clone of Vibrio cholerae and its relationship to other pandemic and epidemic V. cholerae isolates J Bacteriol 1994 176 6199 6206 7928989 10.1128/jb.176.20.6199-6206.1994 PMC196959

[B12] Mutreja A Kim DW Thomson NR Connor TR Lee JH Kariuki S Croucher NJ Choi SY Harris SR Lebens M Evidence for several waves of global transmission in the seventh cholera pandemic Nature 2011 477 462 465 10.1038/nature10392 21866102 PMC3736323

[B13] Lam C Octavia S Reeves P Wang L Lan R Evolution of seventh cholera pandemic and origin of 1991 epidemic, Latin America Emerg Infect Dis 2010 16 1130 1132 10.3201/eid1607.100131 20587187 PMC3321917

[B14] van Belkum A Tracing isolates of bacterial species by multilocus variable number of tandem repeat analysis (MLVA) FEMS Immunol Med Microbiol 2007 49 22 27 10.1111/j.1574-695X.2006.00173.x 17266711

[B15] Stine OC Alam M Tang L Nair GB Siddique AK Faruque SM Huq A Colwell R Sack RB Morris JG Seasonal cholera from multiple small outbreaks, rural Bangladesh Emerg Infect Dis 2008 14 831 833 10.3201/eid1405.071116 18439375 PMC2600222

[B16] Danin-Poleg Y Cohen LA Gancz H Broza YY Goldshmidt H Malul E Valinsky L Lerner L Broza M Kashi Y Vibrio cholerae strain typing and phylogeny study based on simple sequence repeats J Clin Microbiol 2007 45 736 746 10.1128/JCM.01895-06 17182751 PMC1829105

[B17] Grim CJ Hasan NA Taviani E Haley B Chun J Brettin TS Bruce DC Detter JC Han CS Chertkov O Genome sequence of hybrid Vibrio cholerae O1 MJ-1236, B-33, and CIRS101 and comparative genomics with V. cholerae J Bacteriol 2010 192 3524 3533 10.1128/JB.00040-10 20348258 PMC2897672

[B18] Faruque SM Abdul Alim AR Roy SK Khan F Nair GB Sack RB Albert MJ Molecular analysis of rRNA and cholera toxin genes carried by the new epidemic strain of toxigenic Vibrio cholerae O139 synonym Bengal J Clin Microbiol 1994 32 1050 1053 7517950 10.1128/jcm.32.4.1050-1053.1994 PMC267179

[B19] Choi SY Lee JH Jeon YS Lee HR Kim EJ Ansaruzzaman M Bhuiyan NA Endtz HP Niyogi SK Sarkar BL Multilocus variable-number tandem repeat analysis of Vibrio cholerae O1 El Tor strains harbouring classical toxin B J Med Microbiol 2010 59 763 769 10.1099/jmm.0.017939-0 20299504

[B20] Olsen JS Aarskaug T Skogan G Fykse EM Ellingsen AB Blatny JM Evaluation of a highly discriminating multiplex multi-locus variable-number of tandem-repeats (MLVA) analysis for Vibrio cholerae J Microbiol Methods 2009 78 271 285 10.1016/j.mimet.2009.06.011 19555725

[B21] Kendall EA Chowdhury F Begum Y Khan AI Li S Thierer JH Bailey J Kreisel K Tacket CO LaRocque RC Relatedness of Vibrio cholerae O1/O139 isolates from patients and their household contacts, determined by multilocus variable-number tandem-repeat analysis J Bacteriol 2010 192 4367 4376 10.1128/JB.00698-10 20585059 PMC2937383

[B22] Teh CS Chua KH Thong KL Multiple-locus variable-number tandem repeat analysis of Vibrio cholerae in comparison with pulsed field gel electrophoresis and virulotyping J Biomed Biotechnol 2010 2010 817190 20671932 10.1155/2010/817190 PMC2910556

[B23] Feil EJ Li BC Aanensen DM Hanage WP Spratt BG eBURST: inferring patterns of evolutionary descent among clusters of related bacterial genotypes from multilocus sequence typing data J Bacteriol 2004 186 1518 1530 10.1128/JB.186.5.1518-1530.2004 14973027 PMC344416

[B24] Vogler AJ Keys C Nemoto Y Colman RE Jay Z Keim P Effect of repeat copy number on variable-number tandem repeat mutations in Escherichia coli O157:H7 J Bacteriol 2006 188 4253 4263 10.1128/JB.00001-06 16740932 PMC1482962

[B25] Chin CS Sorenson J Harris JB Robins WP Charles RC Jean-Charles RR Bullard J Webster DR Kasarskis A Peluso P The origin of the Haitian cholera outbreak strain N Engl J Med 2011 364 33 42 10.1056/NEJMoa1012928 21142692 PMC3030187

[B26] Ghosh R Nair GB Tang L Morris JG Sharma NC Ballal M Garg P Ramamurthy T Stine OC Epidemiological study of Vibrio cholerae using variable number of tandem repeats FEMS Microbiol Lett 2008 288 196 201 10.1111/j.1574-6968.2008.01352.x 18811655

[B27] Ali A Chen Y Johnson JA Redden E Mayette Y Rashid MH Stine OC Morris JG Recent clonal origin of cholera in haiti Emerg Infect Dis 2011 17 699 701 10.3201/eid1704.101973 21470464 PMC3377427

[B28] Octavia S Lan R Multiple-locus variable-number tandem-repeat analysis of Salmonella enterica serovar Typhi J Clin Microbiol 2009 47 2369 2376 10.1128/JCM.00223-09 19535521 PMC2725683

[B29] Hendriksen RS Price LB Schupp JM Gillece JD Kaas RS Engelthaler DM Bortolaia V Pearson T Waters AE Upadhyay BP Population genetics of Vibrio cholerae from Nepal in 2010: evidence on the origin of the Haitian outbreak MBio 2011 2 e00157 00111 21862630 10.1128/mBio.00157-11 PMC3163938

[B30] Hunter PR Gaston MA Numerical index of the discriminatory ability of typing systems: an application of Simpson's index of diversity J Clin Microbiol 1988 26 2465 2466 3069867 10.1128/jcm.26.11.2465-2466.1988 PMC266921

[B31] Pupo GM Lan R Reeves PR Baverstock PR Population genetics of Escherichia coli in a natural population of native Australian rats Environ Microbiol 2000 2 594 610 10.1046/j.1462-2920.2000.00142.x 11214793

