# Supplementary material for: Multi-locus variable number tandem repeat analysis of 7th pandemic Vibrio cholerae
Source: BMC Microbiol. 2012 May 24;12:82. doi: 10.1186/1471-2180-12-82 (PMC3438101; doi:10.1186/1471-2180-12-82)
Supplement: Additional file 1 — Figure S1.Minimum Spanning trees of 66 V. cholerae isolates using MLVA of A) 6 VNTR loci and B) 4 VNTR loci from chromosome I. Each circle represents a MLVA profile, with the isolate number/s belonging to the MLVA type within the circles. The colour of each circle denotes the group to which each isolate belongs according to SNP typing [12] (see Figure 2). If isolates from different SNP groups shared a MLVA profile, the circle was divided to reflect the proportion of isolates in each SNP group. Thick solid connecting lines represent differences of one repeat unit, thin solid lines and dashed lines represent 1 and 2 loci differences respectively, and longer dashed lines represent more than 2 loci differences. The size of each circle reflects the number of isolates within the circle. [file 1471-2180-12-82-S1.pdf]

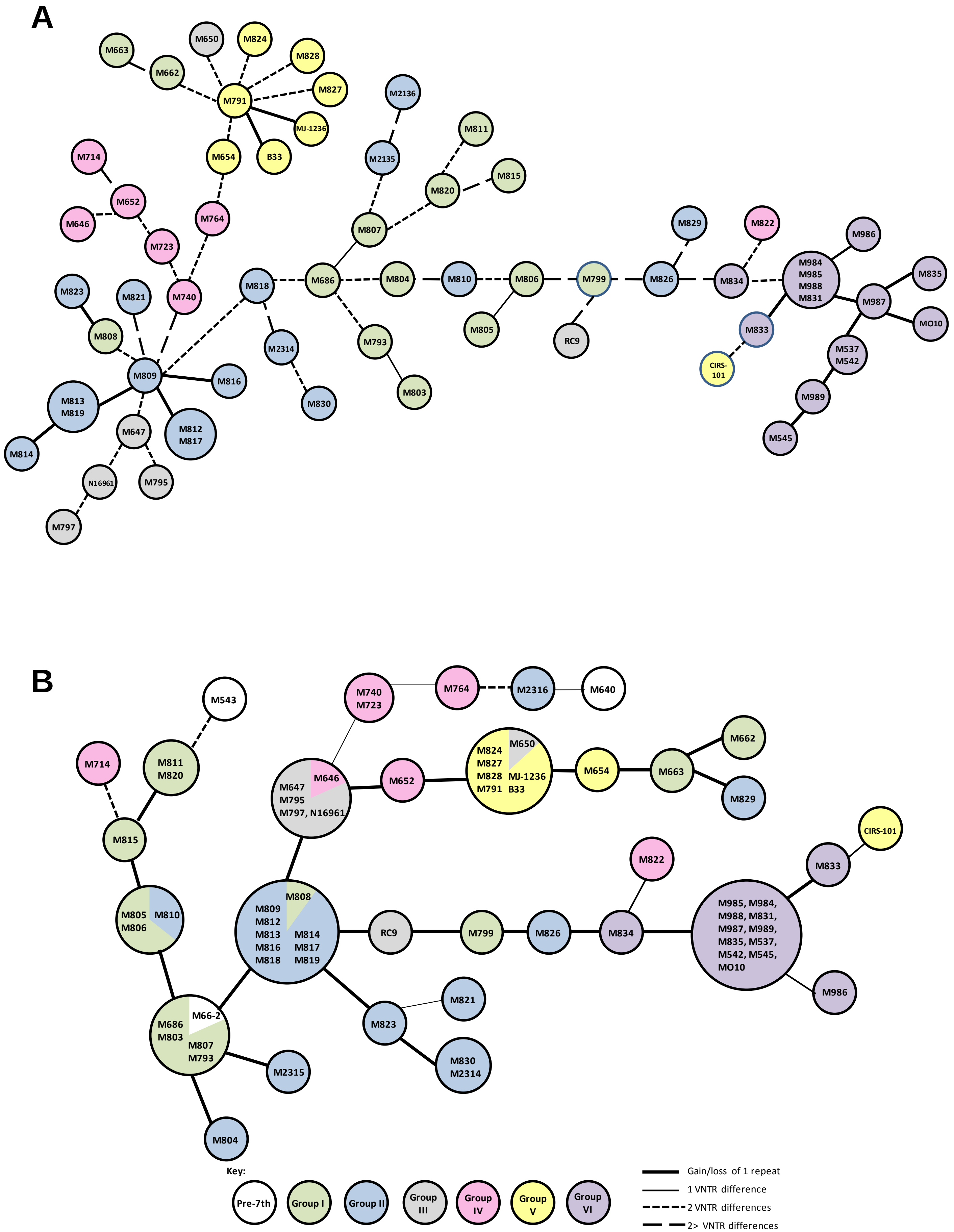

A
M663
M650
M824
M828
M662
M791
M827
M2136
M1-1236
M811
M714
M654
B33
M2135
M815
M652
M820
M646
M764
M723
M807
M829
M822
M986
M823
M821
M740
M818
M686
M804
M810
M806
M799
M826
M834
M984
M985
M988
M831
M835
M987
M808
M805
RC9
M833
MO10
M2314
M793
M537
M542
M809
M816
CIRS-101
M813
M819
M830
M803
M989
M647
M812
M817
M814
M545
N16961
M795
M797
B
M740
M723
M764
M2316
M640
M543
M824
M827
M828
M791
M650
MJ-1236
B33
M654
M714
M811
M820
M647
M795
M797, N16961
M646
M652
M663
M662
M829
M815
CIRS-101
M822
M833
M805
M806
M810
M809
M812
M813
M816
M818
M808
M814
M817
M819
RC9
M799
M826
M834
M985, M984,
M988, M831,
M987, M989,
M835, M537,
M542, M545,
MO10
M986
M686
M803
M66-2
M807
M793
M823
M821
M2315
M830
M2314
M804
Key:
Pre-7th
Group I
Group II
Group III
Group IV
Group V
Group VI
Gain/loss of 1 repeat
1 VNTR difference
2 VNTR differences
2> VNTR differences
